# Supplementary material for: H-theorem in quantum physics
Source: Sci Rep. 2016 Sep 12;6:32815. doi: 10.1038/srep32815 (PMC5018848; doi:10.1038/srep32815)
Supplement: Supplementary Information [file srep32815-s1.pdf]

Supplementary Information for 'H-theorem in quantum physics'

G. B. Lesovik, A. V. Lebedev, I. A. Sadovskyy, M. V. Suslov, V. M. Vinokur

S1. Unitarity constraints

Consider a quantum system (particle) which interacts with a quantum reservoir. The evolution of the joint grand system (particle plus reservoir) can be presented in the form,

$$|\psi_i\rangle|n\rangle \to \sum\nolimits_{jm} U_{jm,in}\,|\psi_j\rangle|m\rangle, \tag{1}$$

where $|\psi_i\rangle$ and $|n\rangle$ are some orthonormal complete sets of states in the particle and reservoir Hilbert space respectively. The transition amplitudes $U_{jm,in}$ comprise a unitary matrix $\hat{U}$: $\hat{U}^\dagger\hat{U} = \hat{U}\hat{U}^\dagger = \hat{1}$.

We assume no energy exchange between the quantum system and the reservoir. Let us consider a fixed energy subspace E of the particle Hilbert space spanned by a basis $|\psi_{i,E}\rangle$, here index i denotes all remaining degrees of freedom of the particle. Then one can represent the grand evolution operator $\hat{U}$ in the form,

$$\hat{U} = \sum\nolimits_E |\psi_{j,E}\rangle\langle\psi_{i,E}|\, s_{ji,E}\hat{F}_{ji,E}, \tag{2}$$

Then the unitarity constraints can be rewritten in the form,

$$\sum\nolimits_i s^*_{ij',E}s_{ij,E}\,\langle m'|\hat{F}^\dagger_{ij',E}\hat{F}_{ij,E}|m\rangle = \delta_{jj'}\delta_{mm'}, \tag{3}$$

$$\sum\nolimits_i s_{ji,E}s^*_{j'i,E}\,\langle m|\hat{F}_{ji,E}\hat{F}^\dagger_{j'i,E}|m'\rangle = \delta_{jj'}\delta_{mm'}. \tag{4}$$

S2. Proof of H-theorem

Here we derive Eq. (3) of the main text and sketch the proof of the H-theorem. We consider a quantum system which interacts with a quantum reservoir. The evolution operator $\hat{U}$ is unitary, $\hat{U}\hat{U}^\dagger = 1$, and using Eq. (2) from the main text we write the unitarity constraints in the form,

$$\sum\nolimits_i s_{ji,E}s^*_{j'i,E}\,\langle m|\hat{F}_{ji,E}\hat{F}^\dagger_{j'i,E}|m'\rangle = \delta_{jj'}\delta_{mm'}. \tag{5}$$

Let the reservoir initially be in a state described by the density matrix $\hat{\pi} = \sum_{nn'}\pi_{nn'}\,|n\rangle\langle n'|$. Then

$$[\Phi(\hat{\rho})]_{jj'} = \sum\nolimits_{ii'}\rho_{ii'}\mathrm{tr}\{\langle\psi_j|\hat{U}|\psi_i\rangle\,\hat{\pi}\,\langle\psi_{i'}|\hat{U}^\dagger|\psi_{j'}\rangle\} \tag{6}$$

where $\hat{\rho} = \sum_{ii'}\rho_{ii'}\,|\psi_i\rangle\langle\psi_{i'}|$.

The system states at different energies are transformed independently. Let us consider a system density matrix at a fixed energy subspace of the particle Hilbert space, $\hat{\rho}_E = \sum_{ij}\rho_{ij,E}\,|\psi_{i,E}\rangle\langle\psi_{j,E}|$. Then making use of the factorization representation of the evolution operator, see Eq. (6), one can rewrite the quantum channel in the form,

$$[\Phi(\hat{\rho}_E)]_{jj'} = \sum\nolimits_{ii'}\rho_{ii',E}\,s_{ji,E}s^*_{j'i',E}\,\langle\hat{F}^\dagger_{j'i',E}\hat{F}_{ji,E}\rangle, \tag{7}$$

where the averaging of the reservoir operators $\hat{F}_{ji,E}$ is done with respect to the density matrix $\hat{\pi}$: $\langle\ldots\rangle = \mathrm{tr}\{\hat{\pi}\ldots\}$. Now let us consider transformation of $\rho_{ii',E} = \delta_{ii'} = \hat{1}_E$. Then using Eq. (7) we find:

$$[\Phi(\hat{1}_E)]_{jj'} = \sum\nolimits_i s_{ji,E}s^*_{j'i,E}\,\langle\hat{F}^\dagger_{j'i,E}\hat{F}_{ji,E}\rangle. \tag{8}$$

Averaging Eq. (5) with respect to the density matrix of the system, moving the left hand side of the averaged equation to the right, and adding the result to the rhs of Eq. (8), one arrives at Eq. (3) of the text:

$$[\Phi(\hat{1}_E)]_{jj'} = \delta_{jj'} + \sum\nolimits_i s_{ji,E}s^*_{j'i,E}\,\langle[\hat{F}^\dagger_{j'i,E},\hat{F}_{ji,E}]\rangle. \tag{9}$$

Now we outline the proof of the H-theorem using Eq. (1) of the main text. To that end we have to calculate $\Phi(\hat{1}) = \Phi(\prod_E \otimes \hat{1}_E)$. Let $[\Phi(\hat{1}_E)]_{jj'} = \delta_{jj'}$ on some subset $\mathscr{E} = \{E\}$. Then using Eq. (8) in the rhs of Eq. (1) of the main text, we obtain

$$-k_\mathrm{B}\sum_{jj',E}\langle\psi_{j,E}|\Phi(\hat{\rho})|\psi_{j',E}\rangle\langle\psi_{j',E}|\log\Phi(\hat{1})|\psi_{j,E}\rangle. \tag{10}$$

Accordingly, in Eq. (10) the terms for which $E \in \mathscr{E}$, become

$$\langle\psi_{j,E}|\Phi(\hat{\rho})|\psi_{j,E}\rangle\langle\psi_{j,E}|\log 1|\psi_{j,E}\rangle = 0.$$

Now if $\langle\psi_{i,E}|\,\hat{\rho}\,|\psi_{i,E}\rangle \neq 0$ only for $E \in \mathscr{E}$ (therefore, for energies $E \notin \mathscr{E}$, all matrix elements $\langle\psi_{i,E}|\,\hat{\rho}\,|\psi_{j,E}\rangle = 0$), the entire expression (10) is zero, Eq. (1) in the main text yields $\Delta S \geqslant 0$, and the theorem is proved.

S3. Example of a non-unital channel

Let us inspect a charged particle moving in a three-lead conductor and interacting with the spin via induced magnetic field, see Fig. 1 in the main text, and, according to the framework of our general consideration, initially disentangled from the spin. In the absence of the external magnetic field the energy exchange is absent and the particle is energy-isolated. The joint scattering states of the particle and spin have the form

$$|\psi_\alpha^{(\mathrm{in})}\rangle|\sigma_0\rangle \to \sum_\beta s_{\beta\alpha}\,|\psi_\beta^{(\mathrm{out})}\rangle\,\hat{U}_\beta\hat{U}_\alpha^\dagger|\sigma_0\rangle, \tag{11}$$

where $|\psi_\alpha^{(\mathrm{in/out})}\rangle$ is the particle's incoming/outgoing state in the lead α, $s_{\beta\alpha}$ are the components of the scattering matrix of the three lead set up, $|\sigma_0\rangle$ is the initial state of the spin and $\hat{U}_\alpha$ ($\hat{U}_\alpha^\dagger$) is the unitary spin-$1/2$ rotation of the spin due to outgoing (incoming) electron in the lead α. Then the operators $\hat{F}$ in Eq. (2) of the main text are defined as $\hat{F}_{\beta\alpha} = \hat{U}_\beta\hat{U}_\alpha^\dagger$.

We choose spin-$1/2$ rotations as $\hat{U}_1 = \hat{1}$, $\hat{U}_2 = i\hat{\sigma}_x$ and $\hat{U}_3 = i\hat{\sigma}_y$, where $\hat{\sigma}_x$ and $\hat{\sigma}_y$ are the Pauli matrices. In this case $[\hat{U}_\alpha, \hat{U}_\beta] \neq 0$. Accordingly, $\langle[\hat{F}_{\beta'\alpha}^\dagger, \hat{F}_{\beta\alpha}]\rangle \neq 0$, and the resulting quantum channel is non-unital. Then,

$$\begin{aligned}\Phi(\hat{1}) = \hat{1} &- \big\{|1\rangle\langle 2|\,2i(s_{11}s_{21}^* + s_{12}s_{22}^*)\,\langle\hat{\sigma}_x\rangle + \mathrm{H.c.}\big\}\\ &- \big\{|1\rangle\langle 3|\,2i(s_{11}s_{31}^* + s_{13}s_{33}^*)\,\langle\hat{\sigma}_y\rangle + \mathrm{H.c.}\big\}\\ &+ \big\{|2\rangle\langle 3|\,2i(s_{22}s_{32}^* + s_{23}s_{33}^*)\,\langle\hat{\sigma}_z\rangle + \mathrm{H.c.}\big\}.\end{aligned} \tag{12}$$

To simplify this equation, we assume time-reversal symmetry of the electron scattering, $s_{ij} = s_{ji}$, and exploiting the following representation[1] of the elastic scattering matrix,

$$s_{ii} = \frac{1}{2}s_{jk}^* s_{ij} s_{ik}\left(\frac{1}{T_{jk}} - \frac{1}{T_{ij}} - \frac{1}{T_{ik}}\right), \quad i \neq j \neq k, \tag{13}$$

where $T_{ij} = |s_{ij}|^2$ are transmission probabilities, we obtain Eq. (5) of the main text.

S4. One-dimensional scattering

From the unitarity of the elastic scattering matrix it follows $|s_{\mathrm{RR}}|^2 + |s_{\mathrm{LR}}|^2 = 1$, $|s_{\mathrm{RL}}|^2 + |s_{\mathrm{LL}}|^2 = 1$, $s_{\mathrm{LL}}^* s_{\mathrm{LR}} + s_{\mathrm{RL}}^* s_{\mathrm{RR}} = 0$, and $s_{\mathrm{RL}} s_{\mathrm{LL}}^* + s_{\mathrm{LR}}^* s_{\mathrm{RR}} = 0$. The unitarity of the joint particle-reservoir gives us

$$s_{\mathrm{LL}}^* s_{\mathrm{LR}}\,\langle n''|n\rangle + s_{\mathrm{RL}}^* s_{\mathrm{RR}}\,\langle n|n'\rangle = 0. \tag{14}$$

Hence $\langle n''|n\rangle = \langle n|n'\rangle$ and

$$s_{\mathrm{LL}}^* s_{\mathrm{RL}}\langle n''|n\rangle + s_{\mathrm{LR}}^* s_{\mathrm{RR}}\langle n|n'\rangle = 0. \tag{15}$$

Let us consider the transformation of the identity operator $\hat{1} = |\mathrm{R}\rangle\langle\mathrm{R}| + |\mathrm{L}\rangle\langle\mathrm{L}|$ if reservoir initially rests in a state $|n\rangle\langle n|$,

$$\begin{aligned}\hat{1} \to\, &|\mathrm{R}\rangle\langle\mathrm{R}|\,(|s_{\mathrm{RL}}|^2 + |s_{\mathrm{RR}}|^2) + |\mathrm{L}\rangle\langle\mathrm{L}|\,(|s_{\mathrm{LL}}|^2 + |s_{\mathrm{LR}}|^2) + \\ &\big[|\mathrm{R}\rangle\langle\mathrm{L}|\,(s_{\mathrm{LL}}^* s_{\mathrm{RL}}\langle n''|n\rangle + s_{\mathrm{LR}}^* s_{\mathrm{RR}}\langle n|n'\rangle) + \mathrm{H.c.}\big].\end{aligned} \tag{16}$$

One can see that the off-diagonal terms $|\mathrm{R}\rangle\langle\mathrm{L}|$ of the transformed identity operator vanish due to unitarity constraint, see Eq. (15), while the diagonal elements sum to the identity operator and hence $\Phi(\hat{1}) = \hat{1}$. The same arguments are valid if initially reservoir rests in an arbitrary mixed state non-entangled with the particle.

The same arguments can be given in a more general form, exploiting the Theorem of the main text. Making use of the scattering states [Eqs. (6)–(7) of the main text] one can construct the joint evolution operator at a given energy of the incoming particle,

$$\begin{aligned}\hat{U} = &\, s_{\mathrm{LR}}|\mathrm{L}\rangle\langle\mathrm{L}| \otimes \hat{1} + s_{\mathrm{RL}}|\mathrm{R}\rangle\langle\mathrm{R}| \otimes \hat{1}\\ &+ s_{\mathrm{RR}}|\mathrm{R}\rangle\langle\mathrm{L}| \otimes \hat{F}_{\mathrm{RR}} + s_{\mathrm{LL}}|\mathrm{L}\rangle\langle\mathrm{R}| \otimes \hat{F}_{\mathrm{LL}},\end{aligned}$$

which transforms the incoming particle states into outgoing states. Here, first two terms corresponds to the forward scattering of the particle which does not change the reservoir state. The last two terms accounts for the backscattering events which do change the reservoir state. The joint unitarity constraint $\hat{U}^\dagger\hat{U} = \hat{1}$ gives,

$$\begin{aligned}&\big(|s_{\mathrm{LR}}|^2\hat{1} + |s_{\mathrm{RR}}|^2\hat{F}_{\mathrm{RR}}^\dagger\hat{F}_{\mathrm{RR}}\big)|\mathrm{L}\rangle\langle\mathrm{L}| + \\ &\big(|s_{\mathrm{RL}}|^2\hat{1} + |s_{\mathrm{LL}}|^2\hat{F}_{\mathrm{LL}}^\dagger\hat{F}_{\mathrm{RR}}\big)|\mathrm{R}\rangle\langle\mathrm{R}| + \\ &\big[\big(s_{\mathrm{LL}}^* s_{\mathrm{LR}}\hat{F}_{\mathrm{LL}}^\dagger + s_{\mathrm{RL}}^* s_{\mathrm{RR}}\hat{F}_{\mathrm{RR}}\big)|\mathrm{R}\rangle\langle\mathrm{L}| + \mathrm{H.c.}\big] = \hat{1}.\end{aligned} \tag{17}$$

The unitarity of the elastic scattering amplitudes $s^*_{\mathrm{RL}} s_{\mathrm{RR}} + s^*_{\mathrm{LL}} s_{\mathrm{LR}} = 0$ vanish the off-diagonal terms $|\mathrm{L}\rangle\langle\mathrm{R}|$ and $|\mathrm{R}\rangle\langle\mathrm{L}|$ on the Eq. (17) if

$$\hat{F}_{\mathrm{RR}} = \hat{F}^{\dagger}_{\mathrm{LL}}, \tag{18}$$

while the normalization condition $|s_{\mathrm{LR}}|^2 + |s_{\mathrm{RR}}|^2 = 1$ and $|s_{\mathrm{RL}}|^2 + |s_{\mathrm{LL}}|^2 = 1$ gives,

$$\hat{F}^{\dagger}_{\mathrm{RR}} \hat{F}_{\mathrm{RR}} = \hat{1}, \quad \hat{F}^{\dagger}_{\mathrm{LL}} \hat{F}_{\mathrm{LL}} = \hat{1} \tag{19}$$

and hence $\hat{F}_{\mathrm{RR}}$ and $\hat{F}_{\mathrm{LL}} = \hat{F}^{\dagger}_{\mathrm{RR}}$ are unitary operators. It follows then that $[\hat{F}_{\beta'\alpha}, \hat{F}_{\beta\alpha}] = 0$ for $\alpha, \beta, \beta' \in \{\mathrm{L}, \mathrm{R}\}$ and the scattering process [Eqs. (6)–(7) of the main text] describes an unital quantum channel.

References

1. Lesovik, G. B., Martin, T. & Blatter, G., Electronic entanglement in the vicinity of a superconductor. *Euro. Phys. B*, **24**, 287–290 (2001).
